# Machine Learning Analysis of Longevity-Associated Gene Expression Landscapes in Mammals

**DOI:** 10.3390/ijms22031073

**Published:** 2021-01-22

**Authors:** Anton Y. Kulaga, Eugen Ursu, Dmitri Toren, Vladyslava Tyshchenko, Rodrigo Guinea, Malvina Pushkova, Vadim E. Fraifeld, Robi Tacutu

**Affiliations:** 1 Systems Biology of Aging Group, Institute of Biochemistry of the Romanian Academy, 060031 Bucharest, Romania; antonkulaga@gmail.com (A.Y.K.); ur.eugen@gmail.com (E.U.); 4mitya@gmail.com (D.T.); lady3mlnm@gmail.com (M.P.); 2 International Longevity Alliance, 92330 Sceaux, France; 3 CellFabrik SRL, 060512 Bucharest, Romania; 4 The Shraga Segal Department of Microbiology, Immunology and Genetics, Faculty of Health Sciences, Center for Multidisciplinary Research on Aging, Ben-Gurion University of the Negev, 8410501 Beer-Sheva, Israel; vadim.fraifeld@gmail.com; 5 SoftServe Inc., 49044 Dnipro, Ukraine; tischenko.vlada@gmail.com; 6 Escuela de Postgrado, Pontificia Universidad Católica del Perú, 15023 San Miguel, Peru; rguinea@pucp.edu.pe

**Keywords:** transcriptomics, cross-species analysis, maximum lifespan, longevity, mammals

## Abstract

One of the important questions in aging research is how differences in transcriptomics are associated with the longevity of various species. Unfortunately, at the level of individual genes, the links between expression in different organs and maximum lifespan (MLS) are yet to be fully understood. Analyses are complicated further by the fact that MLS is highly associated with other confounding factors (metabolic rate, gestation period, body mass, etc.) and that linear models may be limiting. Using gene expression from 41 mammalian species, across five organs, we constructed gene-centric regression models associating gene expression with MLS and other species traits. Additionally, we used SHapley Additive exPlanations and Bayesian networks to investigate the non-linear nature of the interrelations between the genes predicted to be determinants of species MLS. Our results revealed that expression patterns correlate with MLS, some across organs, and others in an organ-specific manner. The combination of methods employed revealed gene signatures formed by only a few genes that are highly predictive towards MLS, which could be used to identify novel longevity regulator candidates in mammals.

## 1. Introduction

Numerous studies have showed that the average lifespan, and in some cases even maximum lifespan (MLS), could be modified by genetic interventions. Hundreds of genes have been shown to be involved in the control of longevity in model organisms or in the etiopathogenesis of aging-related diseases, with many being highly conserved and interacting in a cooperative manner [1,2,3]. Still, until now, only a ~1.5-fold lifespan increase has been achieved through genetic interventions in mammals [4], and even less with pharmacological interventions [5,6]. In contrast, MLS varies in at least a 100-fold range across the Mammalia class [3], hinting that the comparative biology of aging has not been exhausted yet and novel genetic interventions might still be discovered by looking at the differences between various species.

Studying the variations in MLS and transcription across multiple species is an informative method for investigating the evolution of longevity. Recent studies demonstrated that differences in gene expression between long- and short-living mammals exist [7,8,9,10]. In particular, some of the longest-lived mammals display enhanced expression of genes related to DNA maintenance and repair, ubiquitination, immune responses, apoptosis, and autophagy [11]. The overexpression of DNA repair genes is also confirmed by cross-species cell culture profiling [12]. For at least 33 mammalian species the expression of immune response genes in the liver, kidney, and brain shows positive correlations with MLS [9]. There is also clear evidence of pro-longevity transcriptomic adaptations in long-living species such as bats [13], naked mole rats [8,14], and whales [7,11]. 

Many biological processes are non-linear and thus require non-linear techniques for accurate modeling, yet such methods were barely used in comparative transcriptomic studies due to their hard interpretability and data limitations. Advancements in interpretable machine learning brought new explanation techniques for non-linear models, such as, for example, the SHapley Additive exPlanations (SHAP) method, and created an opportunity for the simultaneous characterization of both linear and nonlinear facets of gene expression variability between species. Similarly, Bayesian networks analysis have provided new tools for a more in-depth investigation, allowing us to explore both linear and non-linear dependencies and interrelations between genes, organs, life-history traits and longevity. Moreover, due to rapid developments in next-generation sequencing (NGS) techniques, novel transcriptomes of mammalian species are being added and assembled more and more often, warranting more comparative studies. 

In this study, we aimed to evaluate (i) to what extent the levels of gene expression are linked to mammalian longevity, (ii) if these gene-expression/lifespan associations are organ-specific, and how they relate to life-history traits, (iii) how genes interact with each other in predicting MLS, and (iv) which are the potentially causal genes that might influence MLS. To achieve this, we analyzed gene expression data for five organs (heart, lung, liver, kidney, brain) using linear regression, LightGBM-SHAP, and Bayesian networks. Overall, data from 41 mammalian species were used for these analyses. To draw conclusions, we (i) combined the results from linear organ-specific with nonlinear machine learning models based on decision trees, (ii) used SHAP explanatory models for gene signature prioritization, and (iii) applied a Bayesian networks-based algorithm to select genes which may have potential causality relationships with MLS.

## 2. Results and Discussion

### 2.1. Data Collection and Processing of Gene Expression across Mammalian Species

**Figure 1:**
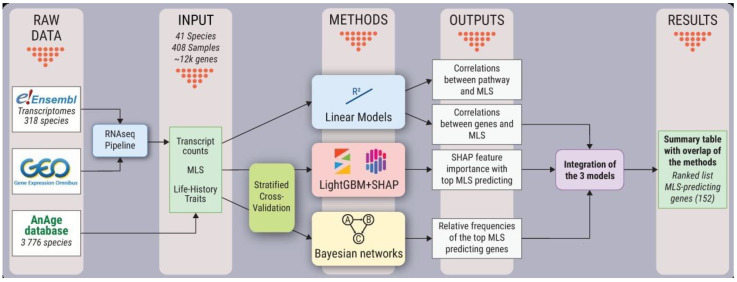
Schematic representation of the analysis workflow used in this study.

Using publicly available RNA-Seq data, we build a cross-species dataset of gene expression levels. The dataset consists of 408 samples from 41 mammalian species and covers five organs: liver, kidney, lung, brain, or heart (the full list of species and sequencing run IDs is available in Supplementary Table S1). The dataset was normalized, processed, and further augmented with species data for studying the associations among gene expression levels and systemic species variables: MLS, body mass, temperature, metabolic rate, gestation period and GC content of mitochondrial DNA, all of which have been suggested to be determinants of MLS [15,16,17]. Linear, LightGBM-SHAP, and Bayesian network models were employed to identify and describe the associations among gene expression levels and MLS (as described in detail in Figure 1). Independent results from three approaches were integrated to investigate which genes will appear as the top MLS predictors, regardless of the methodology differences.

### 2.2. Linear Correlations between Gene Expression and Maximum Lifespan

**Figure 2:**
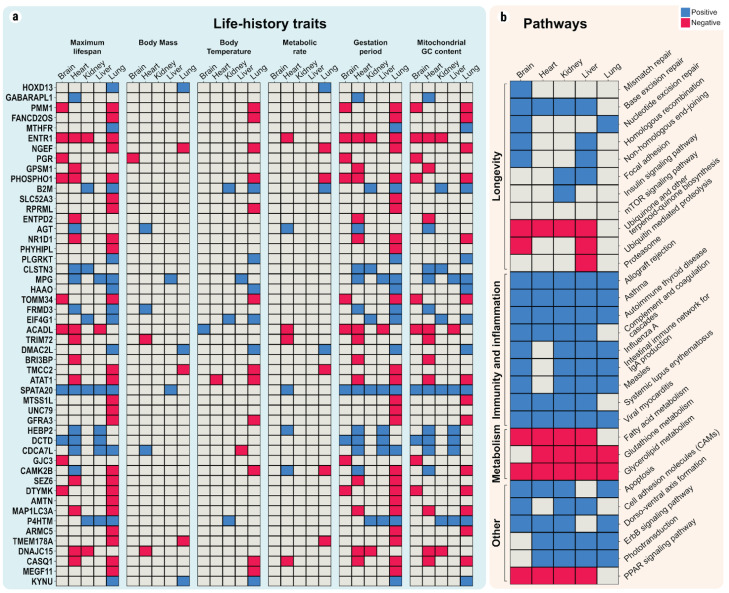
(**a**) Top linear correlations for species features and gene expression. The heatmap represents significant statistical associations (*FDR* < 0.05, *R^2^* > 0.3) between the expression levels of specific genes with MLS and other life-history traits, in different organs. Included are the 50 genes with the highest maximum coefficient of determination (maximum *R^2^* across all organs) for predicting MLS (full list of genes associated with lifespan and other species features in Supplementary Table S2). (**b**) Top linear correlations for MLS and pathway enrichment scores. The heatmap represents the significant associations between MLS and the computed enrichment score (ES) for pathways, obtained using the signature projection approach, which takes into account the expression of all the expressed genes belonging to each pathway. The presented pathways are grouped by categories and include the following: 1) preselected pathways that had been previously shown to be associated with longevity (by independent studies), and 2) additional pathways identified by the current analysis to be associated significantly with MLS in at least four organs. Quantitative details on pathway score associations with other species features can be found in Supplementary Table S3. (**a**,**b**) Red represents significant positive associations for genes or pathways; blue represents significant negative associations. Colorless represents no significant associations found in this study.

To investigate to what extent the expression level of evolutionarily conserved genes correlates with MLS across mammals, linear models were first constructed for 11,831 orthologous genes that are found between 33 mammalian species (Figure 2a). For each of these genes, the coefficient of determination (*R^2^*), which indicates how well the trained linear models explain the MLS variability, was computed. The numbers of genes that were significantly associated with MLS in every organ under analysis are as follows: brain—381, liver—390, kidney—154, heart—535, and lung—756. The median *R^2^* was similar across organs: for brain—0.36, liver—0.36, kidney—0.35, heart—0.38, and lung—0.38. The analysis of the linear models identified that only three genes (CRYGS, TCFL5, SPATA20) have significant positive correlations with MLS (*FDR* < 0.05, *R^2^* > 0.3), in a consistent manner among all five studied organs. It should be noted that the sample size for the heart and lung is relatively lower than that for the other organs, which is mainly due to the generally lower availability for these samples. As such, this bias could be responsible for the small number of genes found to associate in all of the five organs. Consequently, we also looked at the significant correlations that are observable only in the organs with a high sample size: brain, liver, and kidney. The results led to a slightly extended list of 12 genes (SPATA20, TCFL5, TIMP1, HSPB1, RASSF4, SLC25A23, NASP, CCDC14, A2M, NOXA1, C20orf96, CRYGS) whose expression correlates with MLS (*FDR* < 0.05, R^2^ > 0.3) in the brain, liver, and kidney. For a full list of genes associated with MLS and other species’ features, see Supplementary Table S2.

Genes that are predictive for MLS might also correlate with at least one other life-history trait. For example, it is known that MLS correlates with body mass, body temperature, metabolic rate, gestation age, and mitochondrial GC%, which raises the possibility that the associations with MLS are in fact found due to indirect causes. In the brain and kidney, we identified no genes that correlate with MLS uniquely (i.e., genes whose expression correlates with MLS, but not with other variables). In the liver, only one gene (CERS4) correlates with MLS, but not with the other investigated traits. In the heart and lung, we identified 4 and 131 unique associations, respectively, but conclusions drawn from these two organs might be biased because of the lower sample size (we had access to 28 lung samples from 16 species, compared to 121 liver samples from 30 species).

### 2.3. Linear Relationships between Maximum Lifespan and Pathway Enrichment Scores

In order to expand our perspective from individual genes to biological pathways, we used the signature projection approach (ssGSEA) to assess the association between pathway activity (estimated from gene expression) in various organs and MLS (Figure 2b). The ssGSEA method allows for transforming the gene expression space into the biological pathways’ activity space using prior knowledge in the form of gene–pathway association sets. For this, we first selected a list of pathways, such as the mTOR signaling pathway (hsa04150), Insulin signaling pathway (hsa04910), DNA repair pathways (e.g., base excision repair (hsa03410), homologous recombination (hsa03440)), ubiquitin-mediated proteolysis (hsa04120), focal adhesion (hsa04510), etc., which have already been linked to aging or longevity [2,18,19], and then we applied ssGSEA to them.

Interestingly, no significant association was identified for the mTOR pathway, suggesting perhaps that the MLS modulation by mTOR is working amongst the individuals of one species rather than optimizing MLS at the inter-species level, or through other mechanisms such as post-translational modification (e.g., phosphorylation). Contrastingly, for the genes involved in the insulin pathway, we did identify a positive association for the kidney (*p* = 0.005). We also identified multiple strong positive associations of the enrichment score (ES) for several of the DNA repair pathways in the brain: mismatch repair (MMR) (*p* = 3.27 × 10^−5^), nucleotide excision repair (NER) (*p* = 8.35 × 10^−5^), base excision repair (BER) (*p* = 5.46 × 10^−11^), homologous recombination (HR) (*p* = 6.64 × 10^−7^), non-homologous end-joining (NHEJ) (*p* = 4.60 × 10^−8^). Besides the brain, the BER pathway also shows a significant positive association in both liver (*p* = 1.27 × 10^−6^) and kidney (*p* = 0.001), while for the HR pathway the ES is significantly associated only in the liver (*p* = 0.003). For the ubiquitin-mediated proteolysis, we identified a small negative association in liver (*p* = 2.88 × 10^−5^) and brain (*p* = 6 × 10^−4^), but also for the proteasome pathway in liver (*p* = 0.01), whereas for the ubiquinone and other terpenoid–quinone biosynthesis pathways, we found strong negative associations in the liver (*p* = 1.64 × 10^−9^), kidney (*p* = 0.001), brain (*p* = 6.82 × 10^−9^) and heart (*p* = 1.52 × 10^−5^). Focusing on cell adhesion, the focal adhesion pathway displays a small but highly significant positive association, which was detected in the liver (*p* = 1.39 × 10^−5^) and kidney (*p* = 7 × 10^−4^), and for cell adhesion molecules associations were found in the liver (*p* = 9.71 × 10^−7^), brain (*p* = 0.02) and kidney (*p* = 2.70 × 10^−6^). Besides exploring the pathways known to be associated with longevity, we also investigated the pathways that were detected to have significant correlations consistently, i.e., with the same direction of the association, in at least four out of five organs. A set of KEGG pathways involved in infection, inflammation, and the immune response were found to be associated with MLS in multiple organs (Figure 2b), including allograft rejection, asthma, autoimmune thyroid disease, complement and coagulation cascades, influenza A, intestinal immune network for IgA production, measles, systemic lupus erythematosus, and viral myocarditis. To our knowledge, these pathways have not been established as longevity pathways; however, upregulation of the immune response and inflammation with MLS has been shown in both cross-species transcriptomics [9], as well as in studies of long-living animals such as naked mole-rats [10,20]. Enrichment for pathways that are specific to humans (e.g., measles) can be found because of genes from the corresponding gene sets that are involved more generally in inflammation and immunity (e.g., IL-2, IFNα/β). Three pathways involved in metabolism were found to be negatively associated with MLS: fatty acid metabolism, glutathione metabolism, and glycerolipid metabolism. Previous studies have shown a positive association of membrane fatty acid composition [21], and a negative association of glutathione levels in the liver, with lifespan in vertebrates [22]. A negative association of glycerolipid metabolism with lifespan was also found in a cross-species lipidomics study [23]. We also found a negative association for the PPAR signaling pathway. PPARs play an important role in regulating metabolism, and several PPARs display a decreased level with aging, PPAR activity being associated with rising levels of inflammatory mediators during aging [24,25]. Other pathways positively associated with MLS include apoptosis, cell adhesion molecules, dorso–ventral axis formation, ErbB signaling pathway and phototransduction. Some of these have been indirectly linked to health previously; for example, a decreased expression of ErbB signaling in humans is associated with neurodegenerative diseases [26]. This is in agreement with our results, which indicate that increased ErbB signaling is associated with increasing mammalian MLS.

Overall, the linear models in our study emphasize the transcriptomic differences that correlate most with mammalian MLS. The results presented above are generally in line with the findings presented by other cross-species studies, both based on gene expression, but also on metabolomics and lipidomics. In addition to this, we suggest that several pathways, which have been relatively less studied with regard to cross-species lifespan variation, might also play important roles in longevity: PPAR signaling, glutathione metabolism and ErbB signaling. It is important to note, however, that those linear models detect statistical associations that are not necessarily causal for increased longevity. First of all, known or unknown confounding variables could be responsible for the association of expression with MLS. Even if the expression is directly associated to lifespan (without any confounding elements), the results do not allow us to detect whether a pathway is associated with longevity because their components contribute biologically to the increased lifespan (making them “pro-longevity expression traits”), or whether it is associated with MLS due to a deteriorating activity that occurs with aging, and for which longer MLS means longer deterioration (making them “deterioration markers of extended lifespans”). Nevertheless, the detected pathways highlight biological processes whose activities change significantly across the spectrum of mammalian MLS, and which warrant further study in relation to longevity.

### 2.4. SHAP Explanations for Universal Gene Expression Patterns

To also investigate the potentially non-linear patterns of association existing between gene expression and lifespan, we further used an interpretable machine learning approach. Briefly, we applied a gradient boosting decision tree algorithm, using LightGBM [27], to select genes associated with MLS. We then applied SHAP (Shapley additive explanations), a game-theory approach that can be used to explain the output of machine learning models [28], to the LightGBM model, both as part of the selection process and as the main interpretation method. 

Briefly, the key difference between LightGBM and its predecessors is mainly the higher accuracy and computational efficiency. LightGBM has proven (i) to be highly effective for tabular data; (ii) to be a highly explainable model, especially when combined with the SHAP framework; (iii) that it is not sensitive to correlations in the features of the dataset, which is expected for gene expression data; and (iv) that, in comparison to other models (such as neural networks and SVR), it is less prone to overfitting on wide (many features) and small (few samples) datasets.

LightGBM is a non-linear regression model that, once trained, can be used to derive the importance of the employed features (i.e., genes), in predicting the target variable (i.e., MLS), therefore providing means for feature ranking and feature selection. In order to minimize the possibility for selection results to occur by chance, a fold stratified cross-validation procedure is applied and repeated multiple times. Only features selected in all the repeated models were considered further. 

For a particular single prediction, i.e., a sample, a SHAP value for a particular feature (i.e., a gene) represents the impact (i.e., the contribution) of the feature on the model’s predicted MLS within that sample. The SHAP value results from the difference between the prediction when the feature takes a certain value (i.e., the expression in a sample) and the prediction that would be made if the feature took a random value, the latter being an estimation of the average model prediction. One can get a measure of the global feature importance of a gene by aggregating the Shapley values for the gene across all the predictions (i.e., samples).

Initially, we generated the SHAP explanations for life-history traits without including gene-expression data, which showed the high influence of the mitochondrial DNA GC content and of the gestation period on determining MLS (see Supplementary Figure S1). This LightGBM-SHAP model reached a *Huber loss* = 2.19, *MAE* = 2.86, *MSE* = 28.28 and *R^2^* = 0.96 when predicting MLS, with other life-history traits being considered (maximum lifespan, body mass, temperature, metabolic rate, gestation period, and mitochondrial GC%). Of these features, mitochondrial DNA GC content and gestation period were the most impactful traits (Supplementary Figure S1a). This is consistent with previous findings: the gestation period has been linked to senescence [29] and mitochondrial DNA content has been associated with determination of MLS [16]. In addition, several SHAP interaction effects were observed when predicting MLS: between mitochondrial GC content and body mass, between mitochondrial GC content and gestation period, and between body mass and temperature (Supplementary Figure S1b), meaning that these traits conjointly influence MLS variation across species. 

Next, we investigated the effects of various genes whose expression level has an impact on MLS and other life-history traits. For this purpose, we used a two-stage backward feature selection strategy (explained in detail in the methods section). When predicting MLS, the stage II LightGBM-SHAP model used the genes selected by the stage I models as input (155 genes), improving the metrics for MLS prediction from *Huber loss* = 3.92, *MAE* = 4.733, *MSE* = 64.87 and *R^2^* = 0.90 in the stage I model to *Huber loss* = 2.4, *MAE* = 3.04, *MSE* = 36.8 and *R^2^* = 0.95 in the stage II model (outputting 57 genes). Interestingly, several genes included in the stage II model (17 out of 155, slightly enriched, non-significant, *p* = 0.17) are also orthologous to known LAGs recorded in the GenAge database [3]: GNAS, FXN, TERT, MSRA, XRCC6, UQCRB, MEMO1, NEIL1, RPS8, COX7C, RXRB, EIF4EBP1, RCL1, PCBP2, EIF3K, PKN3, CLHC1. 

**Figure 3:**
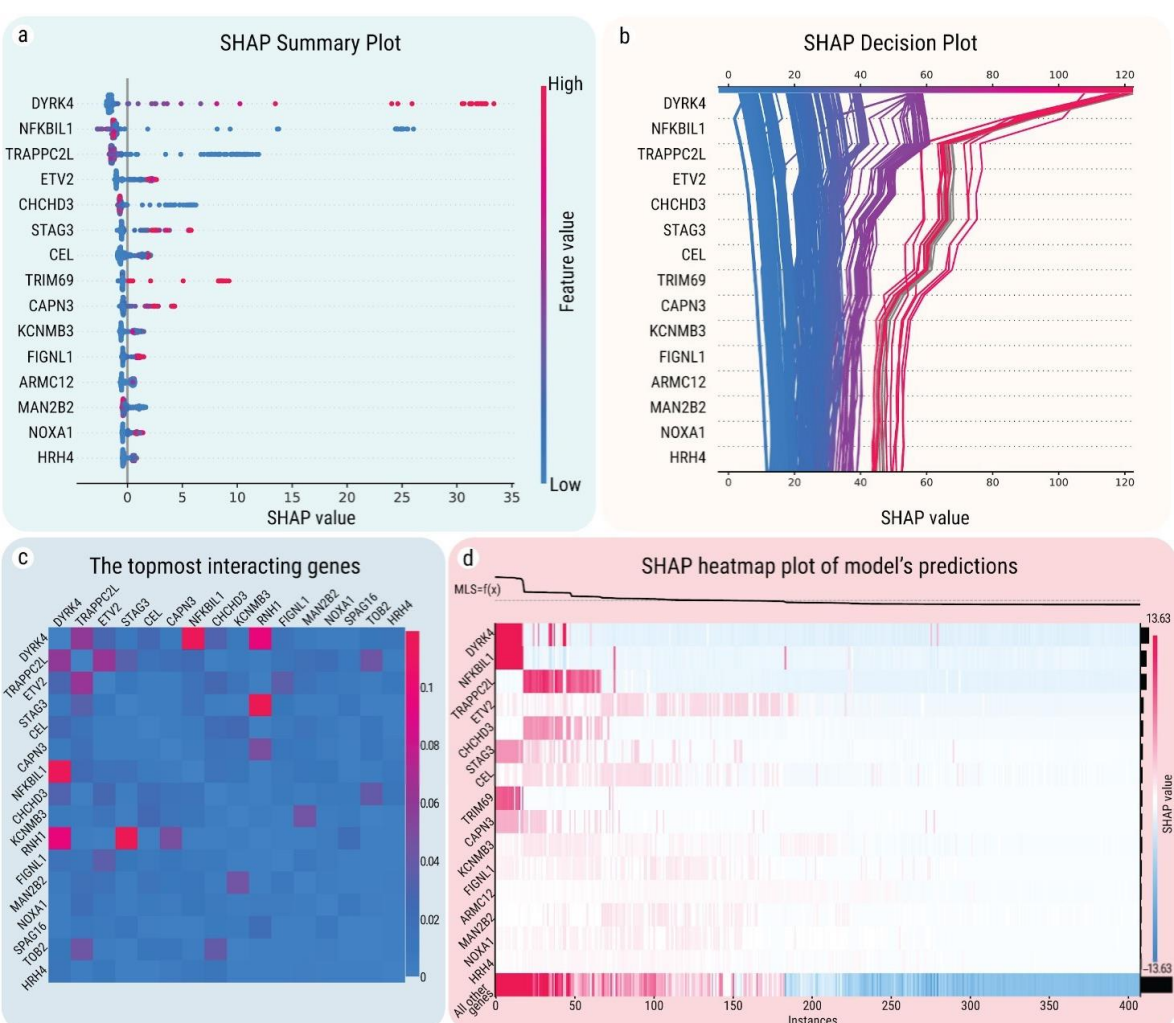
SHAP values and interactions of the top 15 genes with the highest predicted contribution to MLS. (**a**) SHAP summary plot of the impact values for the topmost predictive genes of MLS, based on their gene expression levels. Each dot represents an individual sample in the model, with the horizontal position showing the impact (in years) on the MLS prediction. Colors show the expression of a particular gene in comparison to its baseline expression level across all samples: from blue (lower) to red (higher). Genes are sorted in decreasing order based on the feature importance (mean of the absolute values of impact). (**b**) SHAP decision plot. Each sample’s MLS prediction is represented by a colored line. At the top of the plot, each line intersects the *X*-axis at its corresponding predicted MLS. The color of the fragmented line is defined by the position on the *X*-axis, the predicted MLS (from blue—short-lived species, to red—long-lived species). At the bottom of the plot, the lines will converge at the baseline value (21.6 years), which is an estimation of the average model prediction obtained if all genes used by the stage II model are added. Moving from the bottom of the plot to the top, SHAP values for each feature are added to the model’s baseline value. This shows how each gene contributes to the MLS prediction in a layer-wise, propagating manner. (**c**) The interaction effect (in years) showing stronger or weaker values between pairs of the topmost interacting genes. Each cell depicts the added or subtracted predicted impact (measured in years) that a combination of two genes has, compared to the sum of their individual effects: from blue (lower interaction effect) to red (higher interaction effect). The intensity of the effect (given by the color) does not take into account the direction of the interaction (positive/negative). (**d**) SHAP heatmap plot with the model’s predictions for all samples. Each value on the *X*-axis represents a sample. Genes are displayed on the *Y*-axis, with SHAP values for genes in samples being encoded from blue (lower) to red (higher). The model’s predictions are shown on the top panel (MLS = f(x)), sorted by the MLS value in descending order. The global importance of the genes is shown as black bars on the right side of the figure.

To prioritize genes from the stage II model, we relied on SHAP feature importance (mean absolute contribution) as a way to evaluate the global importance of an individual gene in predicting MLS. That is, for each gene, we have computed the absolute value of SHAP for every gene expression sample, and subsequently, these values have been averaged to obtain the SHAP feature importance. In total, 57 genes that have a mean absolute contribution of more than 0.1 years on MLS prediction were identified. A SHAP summary plot for the top genes which are most predictive for MLS is provided in Figure 3. For many genes, the distribution of SHAP values appears to be skewed to the right (Figure 3a), i.e., the expression of these genes strongly impacts lifespan prediction, positively, in many samples, while most of the genes that impact it negatively are only slightly below zero. In particular, the expressions of the most impactful genes, DYRK4 and NFKBIL1, make a very strong positive contribution (SHAP > +20 in added years) to the MLS prediction for the human samples, as seen in Figure 3b (right-most red trajectories) and Figure 3d (leftmost columns).

Out of the top 15 genes, 5 genes (DYRK4, NFKBIL1, TRAPPC2L, ETV2, CHCHD3) had a significant mean absolute impact (more than 1 year) on the model’s MLS prediction. Moreover, two of these five genes have been previously linked to aging: NFKBIL1 is involved in multiple methylation aging clocks [30], and has been associated with accelerated aging and cellular senescence in studies with genetically engineered mice [31], while CHCHD3 participates in the cross-talk between mitochondrial fusion and the hippo pathway in controlling cell proliferation (apoptosis) [32], which were both found to be involved in longevity in *C. elegans* [33]. STAG3 is essential for maintaining centromere chromatid cohesion and is required for DNA repair and synapsis between homologous chromosomes [34]. It is also known that DYRK4 has different roles in short- and long-living animals. In particular, mouse isoforms of DYRK4 are shorter and expressed mostly in the testis, while human isoforms are longer, expressed in many organs, and differ in localization and substrate specificity [35]. Despite the associations of many DYRKs family genes with neuronal development, Down syndrome, and age-related neurodegenerative diseases [36], DYRK4 still remains understudied in relation to longevity. Besides correlating with MLS, our analysis has shown that these five genes are also predictive for other life-history traits, in particular, DYRK4 and NFKBIL1 are predictive for body mass, DYRK4 and ETV2 for gestation days, TRAPPC2L for mtGC, and NFKBIL1 with CHCHD3 for metabolic rate. To our knowledge, there is no information about the links between TRAPPC2L, ETV2, and aging; however, based on the SHAP analysis, it could be suggested that they might be novel candidates as longevity regulators and should be further investigated.

Calculating the correlation between SHAP values corresponding to gene expression levels, relative to the baseline value (21.6 years; the estimated value obtained if all genes are used), allows for estimating the correlation of the target (i.e., MLS) with the feature (i.e., gene expression), by isolating the effects of other features (genes) on the target. By computing the *Kendall tau-b* ranked correlation coefficient (further denoted as *Kendall tau*), we classified the selected genes with regard to their positive or negative impact on the model prediction (further referred to as pro-MLS and anti-MLS, respectively). NEIL1, NOXRED1, CALCOCO2, CEL, C1orf56 and LRR1 are the strongest pro-MLS genes (*Kendall tau* ≥ 0.6) and C6orf89, PPP1CA, DNAJC15, DPP9 and VARS2 are the strongest anti-MLS genes (*Kendall tau* ≤ −0.6). Of note, the impact direction of the pro- and anti-MLS genes resulting from the LightGBM-SHAP models generally coincides with the ones computed by organ-specific linear models. From the 57 genes selected by the stage II LightGBM-SHAP model, 37 (65%) are found to be significantly associated with MLS (*FDR* < 0.05, *R^2^* > 0.3) by the linear models in at least one organ. The pro-/anti-longevity direction computed as the sign of *Kendall tau* in LightGBM-SHAP coincides for all 37 genes with the sign of the regression slope in the linear models (additionally, the direction is in concordance for significant results in more than one organ, as well). The strongest pro-MLS gene, CALCOCO2, had a positive *Kendall tau* of 0.72 in the LightGBM-SHAP model and was also selected as a pro-MLS gene by the linear models in the lung (*R^2^* = 0.56), brain (*R^2^* = 0.54), liver (*R^2^* = 0.53) and heart (R^2^ = 0.39), while the C6orf89 gene that had a negative *Kendall tau* of –0.79 was selected as anti-MLS by linear models in the heart (*R^2^* = 0.61) and liver (*R^2^* = 0.40). The C6orf89 gene is linked to the NF-κB system [37], whose overactivation is harmful to humans, through a series of age-related processes such as a chronic inflammatory response, increases in apoptotic resistance, a decline in autophagic cleansing, and tissue atrophy [38]. The negative role of PPP1CA in aging has also been recorded, as for instance, it has been found in mice that it plays a role in cognitive aging and its overexpression in cardiac cells resulted in premature heart failure [39]. 

### 2.5. Interactions between MLS-Associated Genes

A gene may impact longevity not only by itself, but also by cooperation with other genes. Based on the cumulative knowledge of interactions between longevity-associated genes (LAGs), it has been previously shown that genes that have a role in determining average or maximum lifespan (as LAGs for example) may have a variety of combined effects—synergistic, additive, dependent, or antagonistic [40]. Thus, the total lifespan changes as a result of genetic interventions targeting two genes are usually not the simple sum of their impacts. To estimate the two-way interactions of the genes selected as potential MLS-associated genes, we also computed SHAP interaction values. The SHAP interaction value of two genes on a sample is the contribution to the prediction of the combined genes after accounting for the contributions of the individual two genes. The matrix shows the strength of interactions, by plotting the difference between the combined SHAP value of a gene pair and the sum of their individual SHAP values effects (depicted by the color intensity in Figure 3c). The results showed that each pair of genes has different effects on MLS prediction. As is shown in Figure 3c, the following gene pairs might have a strong interaction: DYRK4 and NFKBIL1, DYRK4 and RNH1, STAG3 and RNH1, and also TRAPPC2L and ETV2.

We explored the gene pairs with the highest magnitude of interaction (Supplementary Figure S2). DYRK4 is a top gene in terms of overall positive MLS impact; however, when NFKBIL1 is highly expressed, DYRK4 increases MLS to a lower extent (Supplementary Figure S2a). RNH1 (ribonuclease/angiogenin inhibitor 1) is a gene with a high number of interactions. RNH1 is a known regulator of vascularization and a mediator of oxidative stress, which has antioxidant [41] and redox homeostatic [41] effects. As can be seen in the SHAP feature dependency plots, RNH1 has very similar nonlinear interactions with both CAPN3 (Supplementary Figure S2b), DYRK4 (Supplementary Figure S2c), and STAG3 (Supplementary Figure S2d). In all three pairs, the high expression of CAPN3, DYRK4, and STAG3 increases the magnitude of RNH1’s MLS impact in both positive and negative directions, while their low expression values keep the RNH1 impact close to zero. 

### 2.6. Bayesian Networks

In this section, a Bayesian networks approach was used to identify genes that have the potential to be causally associated with MLS while accounting for redundancy and spuriousness. In this context, the potentially causal association of any pair of variables (i.e., gene expression values) was defined as being asserted whenever the correlation between the two holds, regardless of the values others could take (i.e., whenever the two variables do not display conditional independence) [42]. 

For this, first we constructed a Bayesian network, using the genes included in this study and MLS. We employed the notion of a Markov blanket to identify genes that might be causally linked to MLS—the Markov blanket of MLS is the set of genes that are parents, children, or parents of children of the MLS node in the Bayesian network. By definition, the variability in the genes from the Markov blanket of MLS will contain all the useful information about MLS. Subsequently, “potentially causal” gene signatures were defined as gene subsets of the Market blanket of MLS. Implementation-wise, gene signatures were found with SES, a constrained-based variable selection algorithm [43].

While the above-mentioned approach does not fully guarantee causality, it allows a data-driven exploratory analysis to identify valid causal inferences under strict assumptions. In order to give a causal interpretation in an absolute sense, three assumptions would have to hold: the causal Markov assumption, faithfulness, and causal sufficiency [44]. Unfortunately, with only transcriptomics data, which do not include all possible confounders, completely proving causality is not practically achievable, and the employed algorithm deals with these assumptions as best as possible (by construction, the causal Markov condition holds, and using several stratified partitions, the dataset’s underlying conditional independence structure could be approximated, thus attaining faithfulness). Even if causal sufficiency cannot be proven, the results obtained with this method still provide important information about the conditional independence structure between genes and MLS [45].

The Bayesian network analysis included 50 iterations, each corresponding to one stratified train-validation partition, resulting in a set of 50 gene signatures (see Supplementary Table S4 and Figure S3; for methodological details, please see Material and Methods). Supplementary Table S5 shows the relative frequency associated with each gene, representing the proportion of times it was included as part of a signature (i.e., when its *p*-value < 0.01) from the set of all signatures (50). The relative frequencies can be used to rank and therefore prioritize genes with respect to potential causal relations with MLS. Considering all 50 signatures, the most robustly/frequently included genes were the following: NOXA1, C6orf89, NEU2, NDUFA6, RBM46, KCNMB3, and CEL, with relative frequencies of 1.00, 0.94, 0.94, 0.90, 0.82, 0.72, and 0.60, respectively (Supplementary Table S5). 

From a biological point of view, the obtained results are in line with those from the previous section, as NOXA1, C6orf89, and CEL were also identified to be important for MLS determination by LightGBM-SHAP. Additionally, it has been shown that NEU2 upregulation triggers myoblast differentiation in C2C12 cells, and since it is involved in the growth and differentiation of satellite cells, it might implicate the regenerative capabilities of organs such as muscle or heart [46]. NDUFA6 is a key component in Complex I [47,48], which was found to be a biomarker of aging in mice [49] and is downregulated with age in humans [47]. RBM46 might be indirectly linked to aging through its role in the degradation of β-Catenin mRNA in mice [50], and thus through proteasomal degradation in the Wnt/β-signaling pathways. Finally, KCNMB3 has been found to be important for insulin signaling and β-cell function [51]. Taking these genes together and performing a network-based enrichment analysis, it appears that their most enriched biological functions are mitochondria-related, such as the NADH dehydrogenase complex, mitochondrial respiratory chain, mitochondrial organization, and mitochondrial metabolism disease.

### 2.7. Integration of Linear, LightGBM-SHAP, and Bayesian Networks Models

To find the overlaps between the genes predicted by the models employed in this study, we compared the most predictive genes from SHAP explanations, Bayesian networks, and organ-based linear models.

#### 2.7.1. Joint Predictions in Linear and LightGBM-SHAP Models

In most cases, the positive and negative predictive impact of the genes was shared between LightGBM-SHAP (pro- and anti-MLS genes) and linear organ-based models (positively and negatively correlated genes). The pro-MLS genes NOXA1, KCNMB3, CEL, CALCOCO2, LRR1, CAPN3, HRH4, C1orf56 and FIGNL1, which were selected by LightGBM-SHAP, were also selected for different organs by linear models (Supplementary Table S6). Of note, CALCOCO2 and LRR1 have been selected by linear models in all of the organs, hinting that they might be universal determinants. CALCOCO2 (also known as NDP52) is involved in innate immunity and autophagy, which declines with age [52], while Leucine-rich repeat protein 1 (LRR-1) is known to be a determinant of genome stability [53,54]. It is known that FIGNL1 is related to homologous DNA repair [55], HRH4 encodes a histamine receptor that is predominantly expressed in hematopoietic cells and is known to be associated with age-related macular degeneration [56], and KCNMB3 is important for insulin signaling and β-cell function, but its association with glucose-related traits is still unclear [51]. The CAPN3 gene is a major intracellular protease, and some in silico associations with aging exist [57]. NOXA1 is an enhancer for NADPH, which is one of the major reactive oxygen species sources [58], while C1orf56 and CEL are potentially novel LAGs. Several anti-MLS genes in the SHAP analysis were also selected in the linear models, including C6orf89 and DPP9 (Supplementary Table S6). With regards to their functions, it has been shown that C6orf89 exhibits histone deacetylase (HDAC) enhancer properties [59], while DPP9 regulates mitochondrial protein levels and localization [60], and is linked to a variety of age-related pathologies including type 2 diabetes, obesity and cancer.

#### 2.7.2. Joint Predictions in Bayesian and LightGBM-SHAP Models

Genes selected by the Bayesian networks model (the relative frequencies of genes that were part of gene-signatures are provided in the Supplementary Table S5) had high absolute *Kendall tau* values and were also selected by linear models in some of the organs. At the same time, these genes (NOXA1, C6orf89, NEU2, NDUFA6, RBM46, KCNMB3, and CEL) were not the most impactful in terms of mean absolute SHAP values. However, out of them CEL and KCNMB3 belong to the top 10 most impactful genes in terms of SHAP values.

#### 2.7.3. Joint Predictions in All Three Models

Among the genes selected by all models, some strongly correlated with life-history traits and mitochondrial GC content. In particular, the SHAP values of NOXA1 (mitochondria-associated gene) and KCNMB3 strongly and positively correlate with both mitochondrial GC content (NOXA1 *Kendall tau* = 0.65; KCNMB3 *Kendall tau* = 0.62) and gestation period (NOXA1 *Kendall tau* = 0.62; KCNMB3 *Kendall tau* = 0.64). We also observed a positive association between C1orf56 and mitochondrial GC content (*Kendall tau* = 0.6189), and between FIGNL1 and gestation period (*Kendall tau* = 0.41), as well as a negative association between C6orf89 and metabolic rate (*Kendall tau* = −0.57) (Supplementary Table S6).

#### 2.7.4. Composite Ranking

To evaluate the genes selected by different models, the lists of the most predictive genes for linear, tree-based, and Bayesian networks models were combined, and a composite ranking was implemented (Supplementary Table S6). For this, each gene was assigned with a particular rank within each performance metric, and multiple ranks were aggregated (for detailed criteria, please see Methods). 

The composite ranking was then used to determine core gene signatures (as parsimonious as possible in terms of size) with as high an impact on the MLS as possible. Two approaches were used for this—one linear and one non-linear. 

For the linear approach, to study the final selection of genes and their ability to explain the variability in MLS, an organ-wise Bayesian multilevel linear model with random coefficients was used. The top 11 genes were selected based on the penalized deviance criterion. This can be interpreted as “sharing” information across regressions fitted for each organ—a reasonable assumption since we only have so many samples and we can safely assume that information about a particular organ can help us model the regressions done on other organs. As can be seen in Table 1, the genes considered to fit the regression were able to explain more than 70% of the MLS variability for each organ, the brain being the one with the greatest *R^2^* = 0.79 (liver displayed the smallest *R^2^* = 0.71).

**Figure 4:**
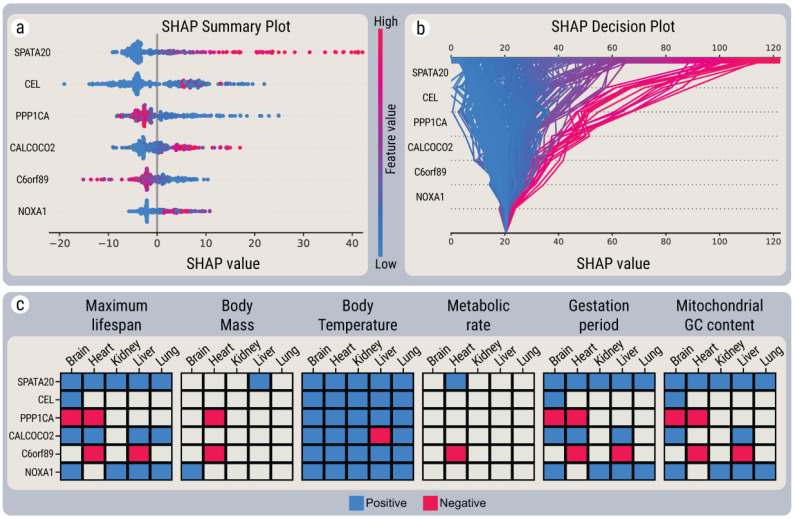
Expression signature of the top six genes in a multi-model analysis. (**a**) SHAP summary plot of the impact of the six most predictive genes of the MLS, based on their expression levels. Each dot represents an individual sample in the model, where its *X*-axis position is an impact (in years) on the MLS prediction of the model in the sample. Colors show the expression of a particular gene in comparison to its baseline expression level across all samples: from blue (lower) to red (higher). The genes are sorted in decreasing order based on the feature importance (average of the absolute value of impacts). (**b**) SHAP decision plot with interactions. Each sample’s MLS prediction is represented by a colored line. At the top of the plot, each line strikes the *X*-axis at its corresponding predicted MLS value, which also defines the color of the line on a spectrum. Unlike in regular decision plot interactions, effects between pairs of genes are included. At the bottom of the plot, the lines approach the base value of 21.3. Moving from the bottom of the plot to the top, SHAP values for each feature are added to the model’s base value of 21.3 years. This shows how each gene contributes to the MLS prediction in a layer-wise propagation manner. (**c**) Linear correlations for species features and gene expressions of the six most predictive genes. The heatmap represents significant statistical associations (*FDR* < 0.05, *R^2^* > 0.3) between the expression levels of the six genes with MLS and other life-history traits, in different organs.

In the non-linear approach, to explain how the genes with the highest composite ranking impact MLS, multiple LightGBM-SHAP models were built with different numbers of top-ranked genes. According to the threshold criteria described in the methods, the top-six genes model was considered most significant, having the lowest number of genes, while having the highest increase in model performance. Upon the training of this stage III LightGBM-SHAP model, average *Huber loss* = 6.41, *MAE* = 7.45, *MSE* = 233.6 and *R^2^* = 0.67 were achieved in a five-fold cross-validation with genes CEL, SPATA20, C6orf89, NOXA1, CALCOCO2 and PPP1CA. Unlike the top genes of the stage II model (Figure 3a), the SHAP distribution of the six genes was more balanced, all genes having both positive and negative SHAP impacts, while better separated clustered samples for pro-MLS (CEL, SPATA20, NOXA1, CALCOCO2) and anti-MLS (C6orf89, PPP1CA) genes could be visually observed (Figure 4a,b). As shown in Figure 4c, the top six genes from the multi-model analysis correlate with systemic features. Remarkably, all six genes, in all five organs, correlate with body temperature, which was previously shown to be an independent determinant of mammalian longevity [17].

## 3. Materials and Methods

### 3.1. Bioinformatic Workflow and Analysis Design

The comparison of expression levels for different species involves tackling many degrees of uncertainty and potential errors due to technical issues (discussed in detail by Toren et al., 2020). With this in mind, we approached the problem as a feature reduction problem, i.e., finding a small set of genes that is highly predictive for MLS variation between species in different organs. The designed bioinformatic pipeline includes processing expression data from multiple species and selecting potential LAGs based on three distinct approaches: linear based models to investigate organ-specific patterns, LightGBM-SHAP explanations models to research the impacts of individual genes and their interactions, and Bayesian networks models to identify potential causality relationships with MLS.

### 3.2. Samples Selection and Data Quality

All mammalian species with transcriptome annotations in the 99th release of the Ensembl Compara Database [61], for which RNA-Seq samples of healthy liver, kidney, lung, brain, or heart organs were available in the NCBI Sequence Read Archive, were selected in this study. The full list of species and sequencing run IDs can be found in Supplementary Table S1. For each species, samples from juvenile, diseased, and very old animals were discarded in order to control for signals pertaining to the developmental or potentially pathological state of the included organisms. Only species that had at least two RNA-Seq samples were kept. In addition, to account for the heterogeneity in expression values, each dataset has been checked for anomalous distributions by exploratory analysis; extreme outliers were manually analyzed and removed. Overall, 408 samples from 38 species and five organs have been selected and processed using the same RNA-Seq quantification pipeline to avoid heterogeneity in the data processing. Linear models were fitted on a reduced dataset in which 5 species were removed: 4 species that contributed with samples to a single organ and 1 species with outlier sample distribution (*Canis lupus familiaris*).

### 3.3. Orthology

Orthology relationships and transcriptome annotations have been obtained from Ensembl, release 99th, preprocessed, and imported to GraphDB, which was further used for the analysis of orthology and for expression value extraction. The preprocessing scripts have been developed in-house and are available as notebooks in our group’s repository at https://github.com/antonkulaga/species-notebooks.

For the linear models, expression values for genes in species with more than one paralog were considered as missing values to reduce ambiguity. The final dataset for linear models included 11,831 genes. For the LightGBM models, 12,323 orthologous coding genes were selected by combining one-to-one and high-confidence one-to-many orthologs. For this analysis, genes for which orthologs were not present in more than 90% of the species were excluded.

### 3.4. Species Life-History Data

Maximum lifespan, body mass, temperature, metabolic rate, and gestation period data have been obtained from the AnAge database, build 14 [3], and mitochondrial GC has been obtained from the MitoAge database, version 1.0 [62].

### 3.5. RNA-Seq Pipeline

For quality control, adapter cutting and trimming, *Fastp* (version 0.20.1) [63] was used. Transcript quantification was done with *salmon* (version 1.4.0) [64]. Transcript expressions were aggregated at the gene level with *tximport* (version 3.12). Raw read counts were normalized on a per-sample basis, using the transcript per million (TPM) normalization. For performing the comparative analysis, the gene expression levels were normalized with TPM, which accounts for potential gene length differences across species.

### 3.6. Linear Models

The linear models’ analysis was performed in Python (version 3.8), using several libraries, including statsmodels (version 0.11.1) [65], pandas (version 1.1.1), seaborn (version 0.10.1), pyUpsetPlots (version 0.4.0), Venn (version 0.1.3), etc. The code necessary to reproduce the analysis can be found at https://github.com/ursueugen/cross-species-linear-models.

To investigate the association between gene expression and MLS, organ-specific linear models were built, allowing for the selection of genes highly associated with MLS, in each organ. Single-variable linear models of the form *GeneExpression ~ β_0_ + β_1_ x SpeciesTrait* were fitted independently for every gene and every life-history trait. For this analysis, the variables were log_2_-transformed and normalized to z-scores, prior to fitting. For each model, missing data (species missing one-to-one ortholog, species with no samples in one of the organs, missing life-history trait) were ignored. Since the number of human samples/data points might bias the analysis and result in much higher leverage for the human species samples (outliers in terms of MLS), we decided to perform the linear analysis while excluding the human samples, since linear regression is sensitive to high leverage points. The *p*-values for *β_1_* were used as the statistical significance of the associations and the signs for the direction of the association, and *R^2^* as the goodness-of-fit metric. All obtained *p*-values were adjusted using the Benjamini–Hochberg multiple testing correction. Associations with adjusted *p*-values < 0.05 and *R^2^* > 0.3 were considered significant. For all results, please see Supplementary Table S2.

The expression activity across pathways was evaluated by using the single-sample gene set enrichment analysis (ssGSEA), also known as the signature projection method, as was first described by Barbie et al. [66]. For pathways data, we used the KEGG pathway database [67]. The signature projection method was applied using the ssgsea function from the *gseapy* Python package (version 0.9.18) [68,69,70]. After obtaining the enrichment score for each pathway, linear models of the form *EnrichmentScore* ~ *β_0_* + *β_1_ x SpeciesTrait* were fitted and interpreted following the same approach as described for single genes. For the full pathway results, please see Supplementary Table S3.

### 3.7. Light GBM Models with SHAP Explanations

To investigate the non-linear patterns of gene expression, we applied a two-stage backward selection with LightGBM and SHAP additive explanations. In the first stage, six separate models were trained, each predicting one of the life-history traits (lifespan, body mass, metabolic rate, temperature, gestation period, and mtGC). For each of the models, the other five species life-history variables were not used in the analysis, such that only genes would provide the prediction power for the target. The union of genes selected by all six models was used as an input for the second stage that made the final selection of the MLS associated genes.

For both stages, the selection procedure involved applying 5-fold cross-validation (CV) with sorted stratification ten times [71]. Sorted stratification was used for achieving similar distributions of MLS in every fold. On each fold, SHAP values were calculated for each gene. The genes that had non-zero SHAP values across all folds were selected (i.e., considered significant), ensuring therefore that the selected genes are resilient to different ways of sample selection and splitting. For each of those genes, we calculated the *Kendall tau-b* rank correlation coefficient between their expressions and the SHAP values across all folds, as a measure of the magnitude and direction of the association between a gene’s expression and the target variable.

It is important to avoid possible bias when the prediction is done solely by the model identifying the species from gene expression. For this reason, data were split into training and validation sets, in such a manner that, on each fold, the validation set contained samples of two species not found in the corresponding training set (unique to every fold).

For each model, we repeated the cross-validation selection ten times, each time with a different random seed. Overall, 10 × 5 = 50 non-unique species pairs were used in the validation across different folds. For each of the 5 models of the first stage, we selected the genes which have non-zero SHAP values in at least two out of the ten cross-validation repeats (i.e., at least 5 × 2 = 10 folds). In the second stage, the selection procedure was made more stringent by selecting genes that have non-zero SHAP values in all the cross-validation iterations/repeats (i.e., 10 × 5 = 50 folds). Through an empirical procedure, the number of top genes to be further characterized was thresholded to 15, corresponding to the elbow of the monotonic graph of SHAP feature importance vs. gene rank (Supplementary Figure S4).

All models were hyper-parametrically optimized in a multi-objective study with the Optuna Framework [72], using maximization of *R^2^*, absolute mean *Kendall tau-b* rank correlation coefficient (between selected gene expressions and their SHAP values), and minimization of *Huber loss* as optimization targets. The parameter set with the best *R^2^* from the Paretto front was selected for the stage I model, and that with the smallest *Huber loss* for the Stage II model. For the stage II optimization, *Huber loss* was prioritized over *R^2^* due to a higher *Kendall tau-b*, combined with a smaller *Huber loss* (which might be caused by *Huber loss*’ resistance to outlier predictions resulting in the selection of genes with better *Kendall tau-b*). A multi-objective tree-structured parzen estimator in Optuna implementation was used to traverse the feature space [73]. The hyper-parametric optimization process was performed independently from the gene selection process. The cross-validation configuration in the processes was similar: 5-fold stratified cross-validation was used in both, except that one fold was excluded in the hyper-parametric optimization (kept as a hold-out and used for evaluating the entire hyperparameter optimization process). Optuna sqlite databases with trials are provided with a source-code repository.

To investigate the effects of gene expressions on MLS and other life-history traits, we used 6 separate regression LightGBM models for predicting from gene expression each of the species’ features: maximum lifespan, body mass, gestation days, temperature, metabolic rate, and mtDNA GC%. For each of the models, the other five species variables were not used in the analysis, such that only genes would provide the prediction power for the target.

### 3.8. Bayesian Networks

A Bayesian network encodes the conditional independence structure among a set of variables, in our case among the input genes and MLS. A potential causal relationship between a gene and MLS is identified when the gene belongs to the Market blanket of MLS in the Bayesian network. This is used as a basis for a variable selection approach that can detect causal relationships under strict assumptions. Implementation-wise, as part of the variable selection methodology, SES, a constraint-based variable selection algorithm [43], was used. This algorithm has proved to be appropriate in the context of high dimensional datasets [43], since the selection is not based on the optimization of an objective function (i.e., loss function), so it is not prone to overfitting. In this study, the MXM R package was used. It generates multiple statistically equivalent parent–children sets (i.e., subsets of the target’s variable Markov blanket). Two of such sets are said to be statistically equivalent if some of their features can be swapped without affecting the inference or the conclusions [43]. Missing values constitute 2.38% in the expression matrix, resulting in 8116 genes and 358 samples to contain at least one missing value. This effect is due to the following: (1) genes being included in the analysis even if they do not have orthologs in 100% of the included species, resulting in missing values for the species without orthologs (see the Orthology section); (2) genes not being expressed at a detectable level in certain organs and thus being flagged by different technologies as missing. In order not to discard a significant amount of samples or genes, a model agnostic algorithm called missForest [74] was used for imputation. This led to missing data being replaced with their estimated values. The aggregated Out of Bag (OOB) NRMSE was 0.33, comparable to errors achieved in other studies [74]. As with the LightGBM-SHAP models, 10 rounds of 5-fold cross-validation with sorted stratification were used, resulting in 50 pairs of training validation sets. For each pair of sets, the SES algorithm used the imputed training set to find at least one gene signature of MLS. For evaluating the performance of each signature, a LightGBM (1800 trees) [75] was fit to the corresponding non-imputed training set and the RMSE between the predictions made using the non-imputed validation set, and the corresponding MLS real values were computed. The signature with the smallest RMSE was saved. Finally, the frequency of the appearance of each gene across all the identified gene signatures was calculated as a measurement of the association between a gene and MLS. The performance of the signature-selection algorithm was measured using the signature RMSE distribution (median = 12.89 years, RMSE range = 6.59–21.58). See Supplementary Figure S3 for the distribution of RMSEs, saved after each iteration of the algorithm.

The fact that some of the signatures from the Bayesian network’s selection (see Supplementary Table S4 and Figure S3) have a big RMSE (i.e., >15 years) should be considered with care, as it does not mean that all the genes within those signatures are unimportant for MLS determination. It is usually the case that some test partitions are “harder” to learn than others.

### 3.9. Integration of Predicted Genes in the Linear, LightGBM-SHAP, and Bayesian Networks Models

Each of the model types (linear, tree-based, and Bayesian networks) resulted in an ordered list of genes, ranked based on various specific metrics. For the tree-based LightGBM model, the metrics were *the number of repeats* (number of rounds when gene mean absolute SHAP value was non-zero), *mean Kendall’s tau* (correlation between gene SHAP values and expression values), and *mean absolute SHAP value* (mean absolute SHAP value of each selected gene across all rounds). For the linear models, selected genes were assigned as a score the *maximal linear R^2^* (maximum *R^2^* across all organ-specific linear models or zero if the gene had no contribution to any linear model). For the Bayesian networks model, the *relative frequency* (i.e., frequency of appearance of a gene across all identified gene signatures) was considered. Constructing the composite ranking included joining all the above-mentioned metrics (*number of repeats, mean Kendall’s tau, mean absolute SHAP value, maximum linear R^2^*, and *relative frequency*), and additionally, *GenAge mentions* were also accounted for for each selected gene (boolean metric: 0 or 1, depending on whether the gene was reported in the GenAge database) [3]. To form a list of genes considered most predictive by all models along with GenAge mentions, the composite rank was computed, as follows: each selected gene was assigned with 6 different ranks, whereby each rank indicated the rank of a gene in a value space of each of the 6 metrics mentioned above. Ranks were calculated with Pandas rank function with the method’s parameter set to dense [76]. Given 6 ranks for each gene, we computed the composite rank of each gene as a sum of its 6 ranks (Supplementary Table S6).

### 3.10. An Explanatory Multilevel Linear Model for Composite Integration

The linear model used for the explanatory analysis of the final selection of genes was a Bayesian organ-wise multilevel model with random coefficients. A normal likelihood, uninformative normal prior for each coefficient, and a gamma uninformative prior for the precision parameter, were used. The regression parameters were fitted using the Gibbs sampling algorithm implemented in the R Package RJags [77]. The ORQ transformation method [78] was used to do a “transform-both-sides” regression with the imputed dataset since the variables (i.e., gene expressions) and the target variable (i.e., MLS) were non-normal. The penalized deviance (2e4 iterations) for the multilevel models with the top 3, 4, 5, 6, 7, 8, 9, 10, 11, 12 and 13 genes was 770.5, 661.7, 639.3, 563.4, 556.3, 545.9, 554.6, 523.8, 491.6, 497.2 and 497.9, respectively. Based on this, the threshold was set to the top 11 genes for the multilevel model. For the single-level model, the penalized deviance was 452.4 for that same selection of genes. The Gibbs sampling algorithm was run using 3 chains, 20e3 iterations, and a burnout period of 1e3 iterations. Finally, for explanatory purposes, we included a column in Table 1 to show the genes that were considered to be significantly involved in the multilevel regression for each organ (i.e., only those whose coefficients’ probability of being greater or less than zero is greater than the threshold 0.70), except for the all-organs regression wherein we include all 11 genes.

### 3.11. Explanatory LightGBM-SHAP Model for Composite Integration

Using the composite ranking and an elbow plot to select the threshold (Supplementary Figure S4), the 15 genes with the highest impact for all models were selected. Multiple LightGBM-SHAP models were then fitted with different numbers of top-ranked genes as features, from top-5 to top-15 genes. For those models, we used the same methodology and species partitioning as in the stage II model. The sequential comparison of the accuracy of those models showed that a significant decrease in *Huber loss* (loss decrease = 0.8) was present during the transition from top-5 to top-6 genes models opposite to a flat decrease after adding more genes to the model (max loss decrease = 0.29). Thus, the top-6 genes model was considered most significant, having the lowest number of genes while having the highest increase in model performance.

## 4. Concluding Remarks

In this work, transcriptomic data from 41 mammalian species were analyzed, using both linear and non-linear organ-specific models. Overall, more than 1800 genes were found to correlate linearly with MLS in at least one of the studied organs. Remarkably, some of these relationships are universal in multiple organs; however, for many other genes, the mechanisms seem to be limited to only some organs. Many of the genes that correlate both with metabolic variables and MLS seem to be expressed in the brain, whereas the liver has the largest number of genes that are associated with MLS independently of other confounders. Pathway enrichment shows that some of the genes found in the analysis are involved in longevity-related biological pathways; however, other pathways, less described or studied so far in relation to aging, also surface and might be of interest.

Using the LightGBM-SHAP and Bayesian networks models, we found gene signatures formed by only a few genes that are highly predictive towards MLS. Interestingly, the genes that we found in signatures are not directly related to each other and belong to different pathways. Remarkably, not many genes that correlate with MLS across mammals are known LAGs, though some LAGs identified in interventional studies do overlap with our results. This is somewhat expected as LAGs are usually found experimentally by knockout or overexpression, and do not necessarily impact species MLS through their expression level. Even so, many of the genes found in our analysis, whose expressions potentially determine mammalian MLS, seem to be directly or indirectly involved in longevity-associated processes. Through a combination of linear, non-linear and Bayesian networks, our analysis highlights novel potential longevity regulators in mammals. This approach could have particular significance for predicting new longevity regulators, analyzing the links between determinants of longevity and associated processes, and studying the mechanisms of aging and longevity.

## Figures and Tables

**Table 1:** Goodness of fit statistics for the multilevel Bayesian linear model.

Organ	Number of Samples	RMSE (Years)	R^2^	MAE	Significant Genes for the Linear Regression
Brain	132	15.47	0.79	8.72	C1orf56, LRR1, C6orf89, CALCOCO2, CEL, DCTD, DNAJC15, PPP1CA, SPATA20, DPP9
Heart	39	12.43	0.78	7.12	C1orf56, C6orf89, CALCOCO2, CEL, DCTD, DNAJC15, SPATA20, DPP9
Kidney	65	11.88	0.73	8.07	C1orf56, C6orf89, CALCOCO2, CEL, DCTD, NOXA1, PPP1CA, SPATA20
Liver	139	8.92	0.71	4.32	C1orf56, LRR1, C6orf89, CALCOCO2, CEL, DCTD, DNAJC15, NOXA1, PPP1CA, SPATA20, DPP9
Lung	33	21.04	0.73	13.40	C1orf56, CALCOCO2, DCTD, NOXA1, SPATA20, DPP9
All organs	408	15.00	0.69	7.90	NOXA1, CEL, CALCOCO2, C6orf89, PPP1CA, SPATA20, DPP9, DCTD, LRR1, DNAJC15, C1orf56

## Data Availability

The preprocessing scripts are available at: https://github.com/antonkulaga/species-notebooks; The bioinformatic WDL pipeline that was used for downloading, quality control and qualification is available at: https://github.com/antonkulaga/rna-seq/tree/master/pipelines/quantification; Code for linear models analysis for genes and pathways is available at: https://github.com/ursueugen/cross-species-linear-models; Code for LightGBM+Shap analysis, the intersection of the results from multiple models and ranking is available at: https://github.com/antonkulaga/yspecies; Code for Bayesian networks analysis and multilevel Bayesian linear modeling available at: https://github.com/rodguinea/bayesian_networks_and_bayesian_linear_modeling.

## Supplementary Materials

The following are available online at https://www.mdpi.com/1422-0067/22/3/1073/s1.
