# Supplementary material for: Machine Learning Analysis of Longevity-Associated Gene Expression Landscapes in Mammals
Source: Int J Mol Sci. 2021 Jan 22;22(3):1073. doi: 10.3390/ijms22031073 (PMC7865694; doi:10.3390/ijms22031073)
Supplement: Supplementary file 1 [file ijms-22-01073-s001.zip › Supplementary/Supplementary Figure legends.docx]

**Supplementary Figure legends**

**Figure S1. SHAP values and SHAP interactions of systemic species traits from the MLS-predicting model**. (**a**) SHAP summary plot for systemic species traits. Each dot represents an individual sample in the model, with the *X*-axis representing the impact (in years) on the MLS prediction. Colors show the expression of a particular gene in comparison to its baseline expression level across all samples: from blue (lower) to red (higher). Traits are sorted in decreasing order based on the feature importance (mean of the absolute values of impacts). (**b**) Systemic species traits interaction matrix. The interaction effect (in years) shows stronger or weaker values between pairs of species features. Each cell depicts the added or subtracted predicted impact (measured in years) that a combination of two species features has, compared to the sum of their individual effects: from blue (lower interaction effect) to red (higher interaction effect). The intensity of the effect (given by the color) does not take into account the direction of the interaction (positive/negative).

**Figure S2. Examples of SHAP feature dependency plots for the top detected interactions**. Gene pairs with the highest magnitude of interaction are shown on **a**–**d**. Each dot represents an individual sample in the model. The feature dependency plots represent the SHAP values of a gene on the *Y*-axis and the gene’s expression levels on the *X*-axis. For visualizing interactions, colors show the expression of a second gene in comparison to its baseline expression level across all samples: from blue (lower) to red (higher). The displayed interactions are between (**a**) DYRK4 and NFKBIL1, (**b**) RNK1 and CAPN3, (**c**) RNK1 and DYRK4, and (**d**) RNK1 and STAG3.

**Figure S3. Histogram of RMSE for Bayesian networks analysis gene signatures.** In each iteration (10 * 5!/4! = 50 in total) at least one gene signature is discovered by the SES algorithm, and the RMSE is calculated by using a LightGBM for each signature and only keeping the smallest one. The bar plot shows how the selected RMSEs are distributed on the real line. The red line represents the median (12.89) of the distribution.

**Figure S4.** Elbow plot for thresholding the number of genes. The blue curve shows the graph of SHAP feature importance vs. gene rank. The orange curve depicts the difference between the SHAP feature importance between two consecutive ranks, which in this case corresponds to the slope. The red vertical line indicates the rank at which the slope (orange curve) becomes negligible and empirically suggests using rank = 15 as a threshold.
